# A synthetic Longitudinal Study dataset for England and Wales

**DOI:** 10.1016/j.dib.2016.08.036

**Published:** 2016-08-26

**Authors:** Adam Dennett, Paul Norman, Nicola Shelton, Rachel Stuchbury

**Affiliations:** a Centre for Advanced Spatial Analysis, University College London, United Kingdom; b School of Geography, University of Leeds, United Kingdom; c Department of Epidemiology and Public Health, University College London, United Kingdom

**Keywords:** Longitudinal, Microdata, Demography, Geography, Social Science, Health Sciences

## Abstract

This article describes the new synthetic England and Wales Longitudinal Study ‘spine’ dataset designed for teaching and experimentation purposes. In the United Kingdom, there exist three Census-based longitudinal micro-datasets, known collectively as the Longitudinal Studies. The England and Wales Longitudinal Study (LS) is a 1% sample of the population of England and Wales (around 500,000 individuals), linking individual person records from the 1971 to 2011 Censuses. The synthetic data presented contains a similar number of individuals to the original data and accurate longitudinal transitions between 2001 and 2011 for key demographic variables, but unlike the original data, is open access.

**Specifications Table**

**Table:** 

Subject area	*Demography, Geography, Health Sciences*
More specific subject area	*Synthetic Longitudinal Microdata Estimation*
Type of data	*Tables, Excel files, R file*
How data was acquired	*Through a synthetic estimation process*
Data format	*Raw*
Experimental factors	*Data were estimated using transitional probabilities derived from the ONS Longitudinal Study for England and Wales and applied to 569,741 individuals contained within the ONS Census Microdata Teaching File*
Experimental features	*Data includes 10 year transitions (2011 back to 2001) for individuals for age, religion, general health, marital status, social grade, live births to mothers and deaths*
Data source location	*England and Wales*
Data accessibility	*Data is within this article*

**Value of the data**

• This data allows students and researchers who are unfamiliar with longitudinal census-based microdata to gain familiarity through hands-on experimentation.
• Data can be accessed without the usual restrictions that are placed on the England and Wales Longitudinal Study.
• This dataset should inspire new substantive pieces of longitudinal research as once more individuals familiar with longitudinal demographic analysis and the research opportunities it presents, then more ideas may flourish.

## 1 Data

The main data file spreadsheet accompanying this article contains 569,741 rows of data (representing 1 individual person per row) with the first 17 columns (in green) containing variables derived from responses to the 2011 Census. The 8 columns immediately following (in yellow) are synthetic longitudinal transition variables estimating the individual׳s state in the 2001 Census. The final two columns contain synthetic estimates of whether the individual would have given birth to children (and how many) or died over the 10-year period. Metadata for all variables are contained in the first two sheets. Supplementary materials also accompanying this article include transitional probability tables for each synthetic variable and R code to generate the synthetic variables from these transitions.

### 1.1 Experimental design, materials and methods

The method we employ is, at its core, a simple one-dimensional proportional fitting exercise making it somewhat more straightforward than the multi-dimensional iterative proportional fitting first proposed by Deming and Stephan [1]. It has been necessary to avoid multi-dimensional variable interactions due to the small cell counts that would occur in the transition matrices.

Our base dataset is the 2011 Census Microdata Teaching File^1^. Transitional probabilities for each variable (for example not married to married or good health to bad health – see Supplementary material) are derived from the LS for a series of 10-year age groups. All transitions are accurate when aggregated to these age groups, although not necessarily when aggregated to another variable such as geographic region.

In the Census Microdata Teaching File, age is recorded for 8 uneven age groups:

0–15
16–24
25–34
35–44
45–54
55–64
65–74
75 and over

These groups are re-estimated so that we have 11 even 10 year groups:

0–9
10–19
20–29
30–39
40–49
50–59
60–69
70–79
80–89
90–99
100+

To carry out the re-estimation to new groups, the single year of age for each person in each original age group is estimated before they can then be allocated a new broad age group. To estimate the single year of age for each of the 569,741 individuals in the dataset, we use data on single year of age for each UK region from the 2011 Census aggregate tables^2^. These Census tables can be aggregated into any age group required and the relative proportions each single age comprises in each group calculated. In doing this, single year of age counts are disaggregated by region due to the large differences in the proportion of the population in each age group in London compared to all other regions in England and Wales.

The total number of individuals of single year of age a in region r will be a fraction of the total number of individuals in age group A in region r:

$$a^{r}\in A^{r}$$

Such that:

$$\sum_{a^{r}=1}^{n}=A^{r}$$

and

$$\sum_{a^{r}=1}^{n}\frac{a^{r}}{A^{r}}=1$$

By calculating all proportions of $\frac{a^{r}}{A^{r}}$ for each age group $A^{r}$ using the Census aggregate tables single year of age file, it is possible to decompose and re-estimate age group data as required.

The estimation of each longitudinal variable transition is carried out in almost exactly the same way for each variable (with some minor variations). Below the general process is described using Approximated Social Grade as the exemplar.

**Stage 1 –** Transitional matrices of the same format are generated for each variable of interest from the ONS Longitudinal Study. These are broadly comparable to the example table below (Table 1) which shows the transitional counts for the Approximated Social Grade variable.

As 2011 is our base population, transitional probabilities are calculated from the counts of transitions with each 2001 state calculated as a proportion of the corresponding 2011 state in turn. Table 2 exemplifies this more clearly:

Taking the first row of Table 1 (Transitions between social grade 1 (AB) in 2011 and social grade 1 in 2001), we can observe that at age group 20–29 (2011 age group), 200 individuals in the LS underwent that transition. Table 2 shows that this is a proportion of 0.045 (4.5%) of all people of social grade 1 at age group 20–29 in 2011 (200/(200+1033+183+3003)=0.045). For each 2011 variable, all 2001 state proportions at each age group will sum to 1. Similar transitional probability tables are generated for each of the variables we transition.

**Stage 2 –** We apply transitional probabilities to the Microdata Teaching File data to create estimates of the total number of people undergoing each transition.

**Stage 3 –** We use the estimates of the total number of people undergoing each transition to update (randomly) the Microdata Teaching File with expected transitions for the correct number of people.

Some small variations in the estimation process were required for variables such as religion and the estimation of births and deaths. For the full estimation process for each variable, see the accompanying processing scripts written in the R language and transitional probability files (which include 2011 to 2001 transitional probabilities for each variable and single year of age counts by region from the 2011 Census).

## Figures and Tables

**Table 1:** Transitional counts between states of Approximated Social Grade, 2011–2001.

**2011**	**2001**	**10–19**	**20–29**	**30–39**	**40–49**	**50–59**	**60–69**	**70–79**	**80–89**	**90–99**
**1**	**1**	0	200	4968	8608	7590	5805	3329	1092	0
**1**	**2**	0	1033	5456	4954	4200	3682	1775	417	0
**1**	**3**	0	183	808	903	695	505	309	87	0
**1**	**4**	0	3003	2250	1089	839	1068	854	232	0
**2**	**1**	11	236	2769	5450	5062	4128	2152	725	0
**2**	**2**	29	1865	8969	11,866	10,954	9233	5668	2090	0
**2**	**3**	10	422	1625	1850	1541	1449	843	218	0
**2**	**4**	60	4732	4159	3722	2828	2785	2227	821	0
**3**	**1**	0	83	669	1303	1260	999	441	114	0
**3**	**2**	0	556	2031	3295	3054	2884	1525	399	0
**3**	**3**	0	805	3107	5318	5225	4029	2478	605	0
**3**	**4**	0	2105	3464	4933	4255	3934	2294	591	0
**4**	**1**	0	95	538	1003	1076	1439	943	313	0
**4**	**2**	10	690	2162	3072	3115	3725	2309	616	0
**4**	**3**	0	411	1403	2281	2452	2844	2262	715	0
**4**	**4**	11	3379	6723	10,162	9864	10,433	9347	3531	11

**Table 2:** Transitional probabilities for Approximated Social Grade, 2011–2001.

**2011**	**2001**	**10–19**	**20–29**	**30–39**	**40–49**	**50–59**	**60–69**	**70–79**	**80–89**	**90–99**
**1**	**1**	0	0.045	0.368	0.553	0.570	0.525	0.531	0.597	0
**1**	**2**	0	0.234	0.405	0.319	0.315	0.333	0.283	0.228	0
**1**	**3**	0	0.041	0.060	0.058	0.052	0.046	0.049	0.048	0
**1**	**4**	0	0.680	0.167	0.070	0.063	0.097	0.136	0.127	0
**2**	**1**	0.1	0.033	0.158	0.238	0.248	0.235	0.198	0.188	0
**2**	**2**	0.264	0.257	0.512	0.518	0.537	0.525	0.520	0.542	0
**2**	**3**	0.091	0.058	0.093	0.081	0.076	0.082	0.077	0.057	0
**2**	**4**	0.545	0.652	0.237	0.163	0.139	0.158	0.204	0.213	0
**3**	**1**	0	0.023	0.072	0.088	0.091	0.084	0.065	0.067	0
**3**	**2**	0	0.157	0.219	0.222	0.221	0.243	0.226	0.233	0
**3**	**3**	0	0.227	0.335	0.358	0.379	0.340	0.368	0.354	0
**3**	**4**	0	0.593	0.374	0.332	0.308	0.332	0.340	0.346	0
**4**	**1**	0	0.021	0.050	0.061	0.065	0.078	0.063	0.060	0
**4**	**2**	0.476	0.151	0.200	0.186	0.189	0.202	0.155	0.119	0
**4**	**3**	0	0.090	0.130	0.138	0.149	0.154	0.152	0.138	0
**4**	**4**	0.524	0.739	0.621	0.615	0.598	0.566	0.629	0.682	1

## Footnotes

1 Available from: http://www.ons.gov.uk/census/2011census/2011censusdata/censusmicrodata/microdatateachingfile.

2 http://www.ons.gov.uk/peoplepopulationandcommunity/populationandmigration/populationestimates/datasets/2011censuspopulationestimatesbysingleyearofageandsexforlocalauthoritiesintheunitedkingdom.

## References

[1] Deming W., Stephan F. (1940). On a least squares adjustment of a sampled frequency table when the expected marginal totals are known. Ann. Math. Stat..

