# Supplementary material for: A synthetic Longitudinal Study dataset for England and Wales
Source: Data Brief. 2016 Aug 26;9:85–9. doi: 10.1016/j.dib.2016.08.036 (PMC5021767; doi:10.1016/j.dib.2016.08.036)

Data In Brief – Conflict of Interest Statement

Adam Dennett

4/8/16

Title: **A Synthetic Longitudinal Study Dataset for England and Wales**

I can confirm that there are no relevant conflicts of interest associated with this manuscript or data

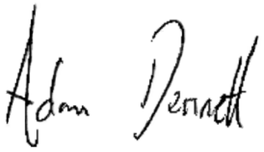

Supplement: Supplementary file 1 — Supplementary material [file mmc1.pdf]
